# Species traits and interaction rules shape a species‐rich seed‐dispersal interaction network

**DOI:** 10.1002/ece3.2865

**Published:** 2017-05-17

**Authors:** Esther Sebastián‐González, Mathias M. Pires, Camila I. Donatti, Paulo R. Guimarães, Rodolfo Dirzo

**Affiliations:** ^1^ Department of BiologyStanford UniversityStanfordCAUSA; ^2^ Departamento de EcologiaUniversidade de São PauloSão PauloBrazil; ^3^ The Betty and Gordon Moore Center for ScienceConservation InternationalArlingtonVAUSA; ^4^ Present address: Department of Applied BiologyMiguel Hernández UniversityElcheSpain

**Keywords:** assembly rules, biological correlates, frugivory, fruit, Pantanal, size

## Abstract

Species phenotypic traits affect the interaction patterns and the organization of seed‐dispersal interaction networks. Understanding the relationship between species characteristics and network structure help us understand the assembly of natural communities and how communities function. Here, we examine how species traits may affect the rules leading to patterns of interaction among plants and fruit‐eating vertebrates. We study a species‐rich seed‐dispersal system using a model selection approach to examine whether the rules underlying network structure are driven by constraints in fruit resource exploitation, by preferential consumption of fruits by the frugivores, or by a combination of both. We performed analyses for the whole system and for bird and mammal assemblages separately, and identified the animal and plant characteristics shaping interaction rules. The structure of the analyzed interaction network was better explained by constraints in resource exploitation in the case of birds and by preferential consumption of fruits with specific traits for mammals. These contrasting results when looking at bird–plant and mammal–plant interactions suggest that the same type of interaction is organized by different processes depending on the assemblage we focus on. Size‐related restrictions of the interacting species (both for mammals and birds) were the most important factors driving the interaction rules. Our results suggest that the structure of seed‐dispersal interaction networks can be explained using species traits and interaction rules related to simple ecological mechanisms.

## 1 Introduction

**Figure 1:**
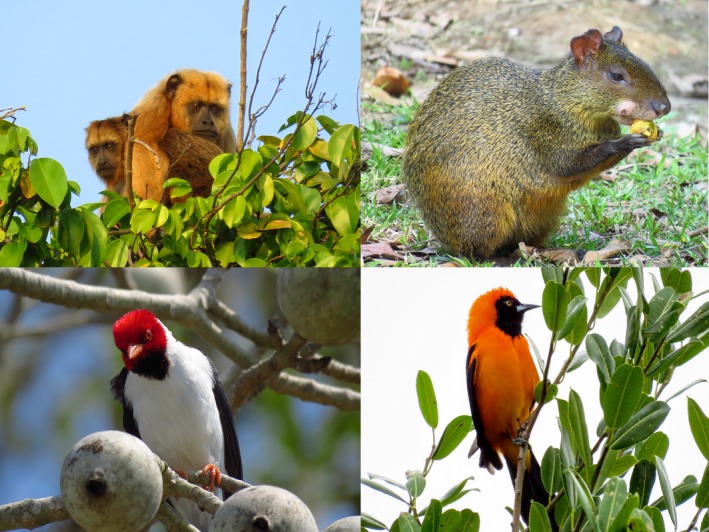
Some of the seed dispersers from the Pantanal community. *Alouatta caraya* (upper left), *Dasyprocta* sp. (upper right), *Paroaria capitata* (lower left), and *Icterus croconotus* (lower right). Photos by Mathias M. Pires

Many fruiting plants, including 50%–75% of the species in tropical floras, depend on animals for the dispersal of their seeds (Figure 1, Jordano & Schupp, 2000). This plant–animal mutualism has important ecological (e.g., plant recruitment patterns) and evolutionary (e.g., fruit size selection) consequences for the interacting species (Galetti et al., 2013; Jordano, 1995). Several studies have found common patterns in the structure of seed‐dispersal networks (Jordano, Bascompte, & Olesen, 2003; Mello et al., 2015; Olesen et al., 2011). For example, some frugivores interact with many plant species while others interact with only a small subset of the plants in the community (Bascompte, Jordano, & Olesen, 2006; Burgos et al., 2007). Plants may also vary in the number of animals that disperse their fruits (Bascompte et al., 2006; Burgos et al., 2007). Other studies have found that seed‐dispersal networks are formed by groups of species that interact more among each other than with species from other groups, the so‐called modular organization (Donatti et al., 2011; Mello et al., 2011). While such patterns can be affected by the environmental context of the network, such as local climatic conditions (Schleuning et al., 2014) or by the scale of the study (e.g., Bezerra, Machado, & Mello, 2009), phenotypic traits of interacting organisms may also play a fundamental role (Vázquez, Blüthgen, Cagnolo, & Chacoff, 2009). Understanding the mechanisms leading to these patterns can be of importance, in turn, to understand the functioning of natural communities.

A salient feature of interaction networks is the fact that some potential interactions never occur; they are the so‐called forbidden interactions (Jordano et al., 2003; Olesen et al., 2011). In seed‐dispersal systems one possible explanation is that some interactions do not occur as a consequence of constraints in the exploitation of the fruits (Burns, 2013; Jordano et al., 2003). In this case, the mechanism behind the absence of the links can be thought as being passive. For example, a frugivorous animal will only be able to eat the fruits (and disperse the seeds) of a plant species if they co‐occur spatially and temporally (Eriksson & Ehrlén, 1991; Olesen et al., 2011). A second possible explanation for missing interactions is a behavioral mechanism related to the preferential foraging for certain fruits. For example, based on Optimal Foraging Theory, animals are expected to forage on food resources that maximize their fitness (MacArthur & Pianka, 1966). Consequently, frugivores are expected to preferentially consume a specific set of fruits that maximize acquired energy relative to the costs of searching and handling, but may also consume other fruits with a decreasing probability (frequency), as the characteristics of the resource deviate from the preferred or optimal (Sobral, Larrinaga, & Guitián, 2010a). A third option is that the network structure is caused both by passive and behavioral mechanisms influencing the interactions at the same time (Santamaría & Rodríguez‐Gironés, 2007). For example, an animal species can prefer fruits with a large energetic content but be only able to consume those that are below a certain size threshold defined by gape width or handling ability. Finally, interaction patterns can also be random, originated by neutral processes (Vázquez et al., 2007, 2009). These different mechanisms shaping network structure define the interaction rules that determine “who disperses who, and who is dispersed by whom” in seed‐dispersal networks.

Several fruit and animal traits may be behind the mechanisms of interaction between frugivores and fruiting plants (Dehling, Jordano, Schaefer, Böhning‐Gaese, & Schleuning, 2016). Size is one of the traits often found to influence interaction patterns for most species (Jordano, 2000; Woodward et al., 2005), and thus potentially also being related to the mechanisms creating the patterns. For example, the relationship between the body size of the consumer and fruit size affect the process of fruit consumption in different ways. Fruit or seed size may passively determine interaction patterns: if beak or mouth gape is not wide enough to swallow the seed or the fruit, the interaction may not take place (Burns, 2013; Jordano, 2000; Wheelwright, 1985). Fruit and seed size may also affect the behavioral decisions of frugivores: large animals may prefer to consume larger fruits maximizing their energetic income (Martinez, Garcia, & Obeso, 2007; Sobral et al., 2010a). Moreover, fruit size has less phylogenetic constraints than other fruit traits (Jordano, 1995), allowing a higher adaptability, and even fast evolutionary changes (e.g., Galetti et al., 2013). Fruit characteristics related to nutritional content may also be important, especially when the mechanisms determining interactions are predominantly behavioral. Fruit energetic content and pulp chemical composition have been shown to affect interaction patterns depending on the frugivore's nutritional requirements (Cazetta, Schaeffer, & Galetti, 2008; Jordano, 1995). Thus, different traits, including morphological features of animals, phenological properties of plants and nutritional content of the fruits may influence the rules that shape seed‐dispersal networks.

In this study, we used three probabilistic models derived from food web analysis to identify the mechanism that explain the interaction rules that organize a plant–frugivore mutualistic assemblage from Pantanal, central South America. To identify the mechanisms at community level, we base our analysis on a tropical, species‐rich system, in which the assemblage of dispersal agents includes both mammals and birds as described by Donatti et al. (2011; see Sarmento, Alves‐Costa, Ayub, & Mello (2014) for another seed‐dispersal assemblage with birds and mammals). Previous work has shown that mammals and birds interact with different partners within this community, leading to semi‐independent groups of interacting species (modules, Donatti et al., 2011). Here we go a step further by exploring the possible mechanisms driving network organization and how these rules depend on the species considered. We specifically aim at identifying if the observed interaction patterns are better explained by (1) a model where interaction patterns are driven by constraints in the exploitation of the resource, thus simulating a scenario where passive mechanisms determine interactions; (2) a model where interactions are biased toward certain combinations of traits, simulating a scenario where behavioral mechanisms determine dietary preferences of consumers; or (3) a combination of both mechanisms. We followed here a “strong inference” approach (Chamberlin, 1890; Elliott & Brook, 2007; Platt, 1964), which consists in comparing multiple working hypotheses. In our case, we compared the three probabilistic models exploring the possible mechanisms driving network organization using information criteria (AIC and BIC, see methods) as mathematical tools to operationalize strong inference. We then test the relationship between the characteristics of the fruits and the frugivores with the interaction rules of these models to examine which possible mechanisms are driving network structure. As the predominant rules and mechanisms may differ depending on the composition of the frugivore assemblage, we performed the analyses (1) for the whole community; and (2) separating the two major frugivore taxa in the network—birds and mammals.

## 2 Methods

### 2.1 Study area and fruit–frugivore interactions

The field data for this study were collected in the Brazilian Pantanal, the world's largest wetland ecosystem, in the Rio Negro (19°34′15″S 56°14′43″W) and Barranco Alto farms (19°34′40″S, 56°09′08″W). Both sites are private areas located close to one another in one of Pantanal's most pristine areas, collectively comprising 18,500 ha. The vegetation consists of a mosaic of seasonally flooded savannah and semi‐deciduous and gallery forests, with average annual precipitation of 1,192.5 mm and mean monthly temperature of 26°C.

We studied a nondefaunated seed‐dispersal assemblage including 45 animal (32 birds, 11 mammals, 1 fish, and 1 reptile) and 46 plant species (see Table S1 in ESM for the complete list of species). The interactions between frugivores and their fruiting plants were recorded using four different methods depending on the frugivore species (Donatti et al., 2011). Seed‐dispersal interactions involving birds were identified through focal observations of 14 plant species (882 hr). Interactions for most mammals, red‐footed tortoises (*Geochelone carbonaria*), and some terrestrial birds were recorded using camera traps (27 plant species, 14,800 hr). This information was complemented with the analyses of 716 scats from mammals, rheas, and red‐footed tortoises. Seed‐dispersal by the pacu fish (*Piaractus mesopotamicus*) was recorded by examining intact seeds found in their intestine (80 pacu fecal samples). This sampling procedure was found to be the most appropriate for a system with such a diverse group of dispersers from different taxa (see “Sampling robustness” section in Donatti et al., 2011). For a more detailed description of the study areas, the basic network organization, and the sampling methods see Donatti et al. (2011).

### 2.2 Interaction rules

We used three probabilistic models with different degrees of complexity derived from food web analyses (Pires & Guimarães, 2013) to identify which assembly rules better describe the interactions between frugivores and fruiting plants: a Cascade model (representing passive mechanisms), a probabilistic Niche model (representing a model where the behavior of consumers has a stronger role) and a Truncated Niche model (representing a combination of both passive and behavioral mechanisms).

**Figure 2:**
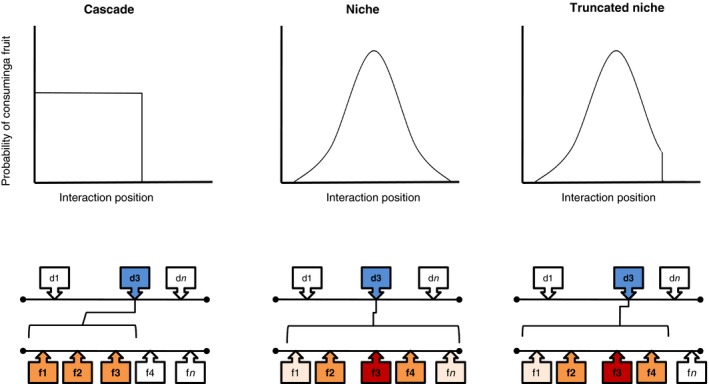
Graphic representation of the three competing models to explain the interaction rules in seed‐dispersal interaction networks. The upper part of each panel shows graphs of the probability that the disperser interacts with a fruit depending on the species position of the fruits (see text for definition). The lower diagrams show a representation of the species position of disperser d3 under each model. In the Cascade model, d3 interacts with f1 to f3 with the same probability, but the probability of interaction with any species after f4 is very low. In the Niche model, the center of the diet is in f3, and d3 will interact with this plant with a higher probability, which decreases as the plant species position deviates from the center. In the truncated Niche model, d3 also has its center in f3 and the probability of interacting decreases while moving away from the center until a threshold is reached, where the interaction probability is very low. Note that the probability of consuming a fruit is never zero in any of the models. However, the value of the probability for the Cascade and the Truncated niche models can be so small (10^−10^) that it is not possible to visualize its difference with the zero value in the *x*‐axis

All models used assume that species can be ordered along axes (one for animals and one for plants) that represent species niche dimensions. Within each axis, species are assigned a *species position* (*n*, see lower diagrams in Figure 2), which is one of the model parameters that define with whom, from the other axis, each species can interact.

In the Cascade model (Cohen, 1990), a species *i* interacts with species *j* depending on whether *n*_*i*_ is larger or smaller than *n*_*j*_. We used a probabilistic version of the Cascade model (Pires & Guimarães, 2013) in which the probability of an interaction between *i* and *j,* given a set of parameters θ = (*n*_1_, *n*_1_…*n*_*S*_, α, β),  is

$$P\left(i,j|\theta \right)\;=\frac{e^{\alpha +\beta \phi_{i,j}}}{1+e^{\alpha +\beta \phi_{i,j}}}$$

The term ϕ_*i*,*j*_ encodes the relationship between *n*_*i*_ and *n*_*j*_ so that ϕ_*i*,*j*_ equals 1 if *n*_*i*_ > *n*_*j*_ or zero otherwise. α and β are parameters that determine how the relationship between *n*_*i*_ and *n*_*j*_ shape interaction probabilities. α determines the probability of interactions when *n*_*i*_ < *n*_*j*_ (ϕ_*i*,*j*_ = 0) and β determines how *P*_*ij*_ changes when *n*_*i*_ > *n*_*j*_ (ϕ_*i*,*j*_ = 1). To be consistent with the original Cascade model α was constrained to be <1 and β is constrained to be >1 so that the probability of interaction is larger when *n*_*i*_ > *n*_*j*_. In this probabilistic version of the Cascade model *P*_*ij*_ is always greater than zero (minimum value 10^−10^) allowing the computation of the model likelihood (see below). The Cascade model can be used to identify a passive mechanism because, for each species, we identify a threshold after which its interaction with the species in the other axis is not possible (i.e., there is a constraint in the interaction).

In the probabilistic Niche model (Williams & Purves, 2011; Williams, Anandanadesan, & Purves, 2010) the diet of the consumer follows the shape of a normal distribution (Figure 2). This means the consumer has a center in its diet (i.e., a preferred item), but a range of possible items may be included in its diet with a lower probability. The probability of interaction between consumer species *i* and resource species *j* is defined by

$$P\left(i,j|\theta \right)=\nu \prod_{d=1}^{D}\text{exp}\left\{-{\left(\frac{n_{d,j}-c_{d,i}}{r_{d,i}/2}\right)}^{\gamma }\right\}$$

where *n*_*d*,*j*_ is the position of the resource *j* on the trait axis, *c*_*d*,*i*_ represents the center of the diet of consumer *i*, and *r*_*d*,*i*_ determines the range of the diet of species *i*. Parameters are defined for each niche dimension of *D* (we use *D *= 1 in this study); γ controls the cut‐off rate of the probability function and ν is the maximum probability of interaction (here, ν = 1). Therefore, the rules of the Niche model are related to more behavioral mechanisms and can be related to Optimal Foraging Theory predictions, which propose that diet is determined by foraging decisions that depend on characteristics of the resource and the consumer.

We also propose a third model, the Truncated Niche model, resulting from the combination of the rules from Niche and Cascade models. In this model (Figure 2), the probability that *i* and *j* interact is defined in the same way as in the Niche model, until it reaches a threshold defined by the relationship between *n*_*i*_ and *n*_*j*_. Thus, for *n*_*j*_ > *n*_*i*_ the probability drops, approaching zero.

The *species position* (*n*) of each of the animal and plant species on the axes are free parameters in the models and are estimated using empirical data on interactions. We initially assigned species positions and other model parameters (see below) randomly and then used an optimization algorithm, the simulated annealing (Kirkpatrick, Gelatt, & Vecchi, 1983; original code from Vandekerckhove, 2008), to find the parameter set that maximizes the likelihood for each model (see description below). We used the Latin hypercube (Mckay, Beckman, & Conover, 1979) to explore large parameter space in the initial conditions of the optimization through simulated annealing.

To compare model performance in reproducing the network structure we computed the model log‐likelihood of each matrix *A* representing the interaction network as:

$$L\left(\theta /A\right)=\sum_{i}^{}\sum_{j}^{}\ln\left\{\begin{array}{cc} P(i,j|\theta ) & \text{if}\;a_{ij}=1\\ 1-P(i,j|\theta ) & \text{if}\;a_{ij}=0 \end{array}\right\}$$

As the number of parameters of the models differs, we compared the models using information criteria that identify the most parsimonious models (i.e., those that have the highest likelihood with the lowest number of parameters). We used the Akaike Information Criterion (AIC) and the Bayesian Information Criterion (BIC). The BIC introduces a higher penalty than the AIC (i.e., it has a higher penalization of models with large number of parameters). Thus, to allow comparisons with other studies, we discuss the results based on the AIC, which is the standard method used for model selection based on the likelihood approach, but we also report the BIC values. To establish a reference point for the goodness‐of‐fit measures, we also calculated the fit of a random model in which all interactions are in principle possible with the same probability and with no systematic constraint on interactions. In this model *P*_*ij*_ = number of interactions/(number of animals × number of plants). We repeated the optimization procedure estimations 30 times per model, starting from different points in the parameter space in order to have more reliable estimates of the parameters. All the models were implemented in MATLAB (2012). Codes for this modeling are available as Appendix S1 in ESM.

As the interaction rules for mammals and birds may be different because of differences in body size and their preferences for different fruit traits (Jordano, 1995), we first ran the model selection procedure for the whole assemblage, and then re‐ran the analysis separately for birds and for mammals, without including the fish species and the tortoise species in this set of runs. Moreover, the original interaction network in Donatti et al. (2011) includes the non‐native feral pig (*Sus scrofa*). However, we did not include this species in our analysis because we wanted to make the results transferable to other sites in the tropics where there are no exotic species that could potentially take over seed‐dispersal services of locally extinct native species.

### 2.3 Animal and plant traits

We gathered information on the traits of plants and animals to identify the potential biological drivers behind the network interaction rules. For each species of plants, we used the information in Donatti et al. (2011) and Donatti (2011) that includes diameter (in mm) and mass (in g) of fruits and seeds, protein content (mg), carbohydrates and lipids (per gram of dry fruit), the energy content of fruit (kcal/gram of dry pulp), and fruit availability (measured as density of a plant species/ha × average number of months with mature fruits/year × average number of fruits produced/individual plant). For the frugivores the information included the log‐transformed body mass (kg), the degree of frugivory of the species (from 1: hardly ever consumes fruit, to 5: exclusive frugivore), and beak width or mouth size (mm). We obtained the information on beak width for the majority of bird species and bird degree of frugivory from the literature (Del Hoyo, Elliot, & Sargatal, 1992). For mammal mouth size, and for some birds, one of us (ESG) measured the skull (for mammals) and the beak (for birds) of specimens (2‐12 individual birds) deposited at MVZ (Museum of Vertebrate Zoology), University of California Berkeley (see Table S2). For birds, we measured beak width at the base of the closed beak, while for mammals we measured mouth size as the space between the last teeth of the inferior jaw.

### 2.4 Relating model parameters with species traits

To identify which biological attributes perform better in explaining the interaction rules identified with model selection, we examined the relationship between the values of model parameters obtained after optimization and the animal and plant traits described above, using generalized linear models (GLM). We performed all analyses in R (R Developmental Core Team, 2014) using GLMs with a Gaussian distribution. As dependent variables we used the species position (*n*_*i*_) when the interaction rules followed the Cascade model by means of AIC, and the interaction center (*c*_*d*,*i*_) and range (*r*_*d*,*i*_) when they followed the Niche model. For plants, we used the species position (*n*_*j*_), which is the only parameter related to the resources in all models. As explanatory variables, we used all the plant and animal traits described in the section *animal and plant traits*. We evaluated the significance of the variables in question by a random permutation of the dependent variables (i.e., model parameters values) and re‐running GLM tests using the randomized variables. We repeated this procedure 1000 times and checked if the GLM coefficient estimates were within or outside the 95% confidence interval of the distribution of the same coefficients based on the randomizations. We used univariate models in all analyses (i.e., one predictor variable). R codes for this GLM analysis are available as Appendix S2 in ESM.

As a validation procedure, we also ran the model selection routine while considering the information on species traits. Instead of parameterizing the model using the interaction data, which would impose a certain functional relationship between model parameters and traits, we used trait values to rank species and, at each iteration of the optimization used to find the maximum likelihood estimates (MLEs), we reassigned parameter values according to the trait rank. For example, to test how good was animal body mass in describing the interaction patterns, we ranked the species according to body mass and we kept this order when assigning the species position in the Cascade Model or the parameters of the Niche Model in the simulations, so that the animal with largest body mass always had the largest *n*_*i*_ (or *c*_*i*_ or *r*_*i*_) and so on. The advantage of this procedure is that we do not make any assumption on the specific relationship between model parameter and traits, except that there is a correlation between them. We performed simulations constraining the distribution of parameters (i.e., *n*_*i*_, *c*_*i,*_ and *r*_*i*_), one at each time, and all combinations of constrained and unconstrained parameter distributions. We obtained MLEs using simulated annealing and used the AIC to compare the fit of the models. These models constrained by species traits are expected to perform worse than the unconstrained model, because the latter allows any parameter distribution. However, by comparing the fit of the different constrained models we can tell whether there are traits that better predict the observed network structure than others. We could only perform these simulations for those species traits that did not have missing values. This included body mass and the degree of frugivorous diet for animals, and seed and fruit size for plants.

Finally, we wanted to gauge if we could reproduce our interaction network using the interaction rules. To do so, we use the MLEs to create a matrix with the probability for each interaction to occur. Using these probabilities, we created 1000 random matrices and we compared each of them with our empirical interaction network. For each matrix, we calculated the proportion of correctly predicted interactions (both presence and absence of interactions) as the total number of interactions in the simulated matrix that were equal to the interactions in the empirical matrix, divided by the total number of possible interactions (number of plants × number of animals). Then, we calculated the average proportion of correctly predicted interactions in the 1000 matrices. Finally, we also calculated for each species the proportion of simulations where its interactions were correctly predicted by the model. We performed this analysis for the complete matrix, and for matrices with only birds and only mammals.

## 3 Results

### 3.1 Interaction rules

Of the three models considered, the Cascade model outperformed the other models in reproducing the structure of the analyzed seed‐dispersal interaction network (Table 1), suggesting that interaction patterns for the seed‐dispersal system as a whole are better described by constraints in the exploitation of the fruit resources (i.e., the passive mechanism). The Cascade model was also selected when we compared the fit of the models for the bird species. In contrast, mammal interaction patterns were better reproduced under the assumptions of the Niche model (Table 1). These results suggest that the interaction patterns between mammals and fruiting plants are better explained by interaction rules that allow species preferences related to one or a few correlated fruit characteristics (i.e., the behavioral mechanism). The results were slightly different using a more conservative goodness‐of‐fit measure (BIC). For mammals, the likelihood of the Cascade and Niche models did not differ significantly under BIC. No matter the metric used, however, the random model for the matrix with both birds and mammals had a worse fit when compared to the other models (ΔAIC = 548.0).

**Table 1:** Likelihood values (AIC and BIC) for each of the three competing models considering: the complete assemblage (“All”), mammals only, and birds only

Group	Metric	Cascade	Niche	Truncated Niche
All	AIC	**1116**	1126	1138
	BIC	**1452**	1725	2001
Birds	AIC	**514**	530	556
	BIC	**734**	842	960
Mammals	AIC	394	**368**	380
	BIC	**550**	**552**	619

The model selected for each simulation is shown in bold.

Next, we used the MLEs of model parameters obtained from optimization to generate theoretical networks and test whether the models are indeed capable of reproducing the empirical interaction network. The models accurately predicted an average of 86.6% (±0.6) of the interactions (both the presence and the absence of interactions) in the matrix including all the species. Moreover, the mean percentage of correctly predicted links at species level when analyzing the network for the birds was similar to the mean proportion of correctly predicted links when analyzing the matrix with only the bird species (91.2 ± 6.5 for entire network vs. 91.1% ± 0.5 for the analyses with only birds). However, the analyses including only mammals and under the premises of the Niche instead of the Cascade model, increased the mean proportion of correctly predicted links for the mammal species (74.4 ± 13.5 entire network vs. 84.8% ± 1.2 analyses with only mammals). Also, the proportion of correctly predicted links increased with seed diameter for the entire matrix and for the matrix with only birds (GLMs, both *p *< .001, Fig. S1 in ESM), but not for the matrix with only mammals, which fitted better a polynomial distribution (Fig. S1). Finally, the proportion of correctly predicted interactions was lower for those species with more interactions (GLMs, all *p *< .001, Fig. S2).

### 3.2 Traits underlying the interaction rules

**Figure 3:**
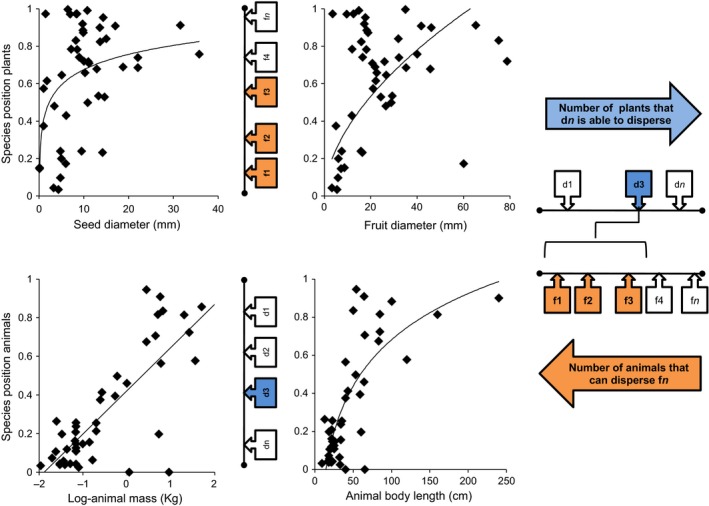
Representation of the significant relationships between plant or animal species trait variation and their species position from the model selected (in this case, the Cascade model) using the AIC approach. Relationships are shown for the complete community (including both birds and mammals). The sketch shows how the species position should be interpreted: Animals with a high species position in the axis will be able to disperse more fruits, while fruits with a high species position in the axis will be dispersed by fewer animals. We also show the best fit for the data: Logarithmic for seed diameter and body length, power for fruit diameter, and linear for body mass

**Figure 4:**
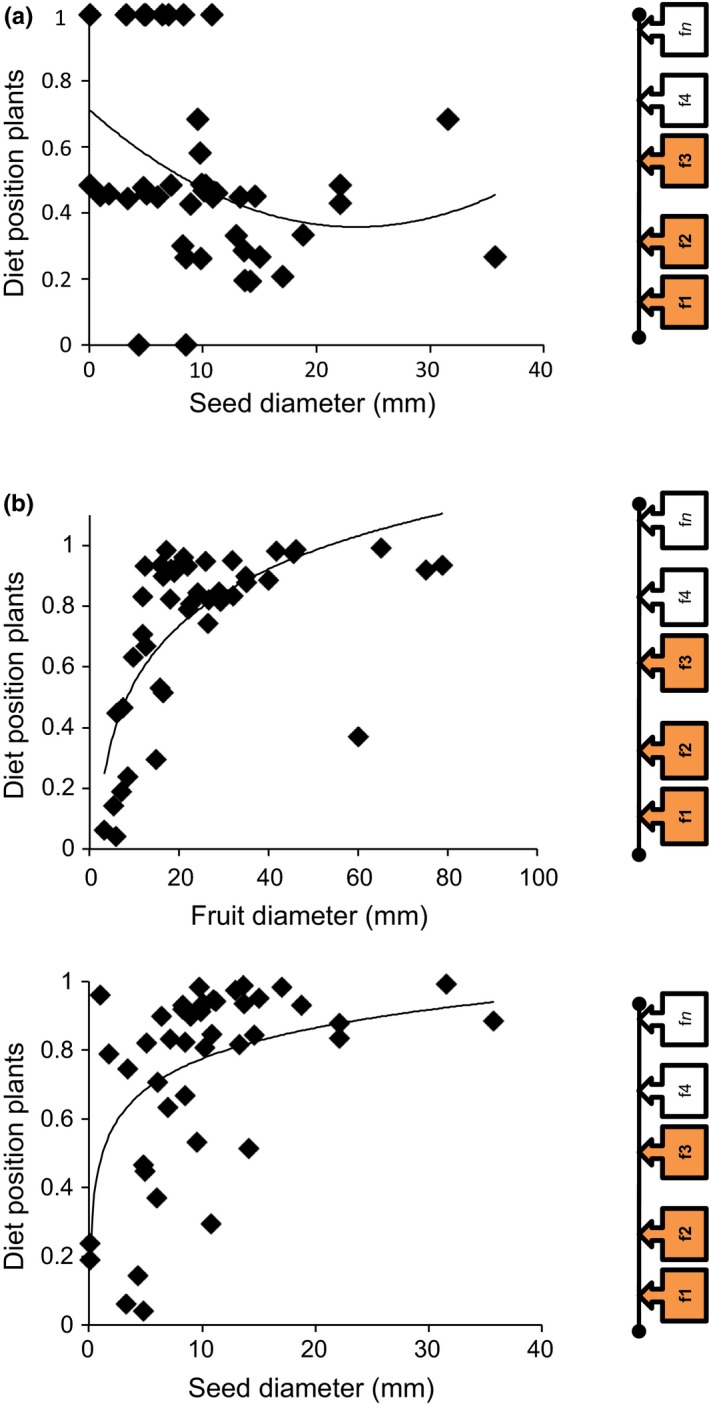
Representation of the significant relationships between plant or animal species trait variation and their species position from the model selected using the AIC approach for mammals (a) and birds (b). No animal biological characteristics were significantly related to species position. See Figure 2 for a sketch explaining how the species position should be interpreted: Animals with a high species position will be able to disperse more fruits, while fruits with a high species position will be dispersed by less animals. We also show the best fit for the data, which was polynomial for mammals and logarithmic for birds. The model selected was the Cascade for birds and the Niche for mammals

GLMs indicated that the interaction rules are mainly driven by fruit, seed, and animal sizes (Table 2). When we analyzed the entire community (Figure 3, Table 2), larger seeds and fruits were associated with higher parameter values (*n*_*i*_), which translates in being dispersed by a contingent of fewer species, corresponding to large‐bodied animals. Accordingly, dispersers with small body size and body length presented lower values for model parameters*,* being able to disperse a smaller contingent of plant species. When we examined birds and mammals separately, we found that none of the animal characteristics analyzed correlated with the model parameters corresponding to each species, yet both fruit and seed size for the bird network and fruit size for the mammal network were important drivers of the interaction patterns (Figure 4, Table 2). Interestingly, the relationship between parameter values (*n*_*i*_ or *c*_*i*_) and fruit size was different for the bird and mammal networks. While birds dispersed preferentially small fruits (i.e., *n* and fruit size were positively related), mammals dispersed preferentially the largest ones (i.e., *c* and size were negatively related).

**Table 2:** Sign and significance of the generalized linear models relating interaction position (*n*_*i*_), interaction center (*c*_*i*_), or interaction range (*r*_*i*_) of the plants and the animals, with their biological characteristics

	Plant position		Animal interaction center	
	Fruit diameter	Seed diameter	Log‐body mass	Body length
Complete	+*	+***	+***	+***
Birds	+*	+**		
Mammals		−**		

When the model selected was the Cascade, there was only one position value that corresponds to the threshold that separates fruits that can be or cannot be consumed by each species. When the model selected was Niche, the diet of the animal was given by the center of the interaction. We also tested the relationship with the range of the interaction, but none of the models were significant.

Significance as follows: *p < .05, **p < .01, ***p < .001.

The results were similar for the analyses where model parameters were constrained by species traits (Tables S3–S5). For both the entire community and birds, the best fit was obtained when constraining the position of the plants in the cascade model using fruit diameter (Tables S3, S4). When we considered only mammals, constraining the location of the species position for animals by body mass using the cascade model (and not the niche, as for the unconstrained model) resulted in the best fit (Table S5).

## 4 Discussion

There is an ongoing discussion regarding the prevalence of passive vs. behavioral interaction rules in determining mutualistic interaction patterns, with some studies supporting preferential foraging (Moermond & Denslow, 1985; Tewksbury & Nabhan, 2001), and others supporting random and interchangeable interaction patterns only limited by trait‐based constraints (Burns, 2006, 2013; Zamora, 2000). However, most of these studies focus only on bird species. In this study, we combined high‐quality field data with a model selection approach to infer the possible rules that drive plant–seed disperser interaction patterns in a species‐rich ecological community of bird and mammal dispersal agents. Our results show that, while interaction patterns for birds are better described by passive rules driven by morphological constraints, mammal interactions are better reproduced by rules that include a stronger behavioral component representing variations in animal preferences according to fruit traits. We also identified that these rules are mainly driven by differences in the size of the fruits, seeds, and the body size of the frugivores.

The contrasting interaction mechanisms acting in the birds and mammals networks seem to be related to the large differences in size of the two animal groups. Trait‐based filtering has been identified as one of the main processes organizing different types of plant–animal mutualistic interactions (Schleuning, Fründ, & García, 2015). From the different traits affecting species interactions (Vidal, Pires, & Guimarães, 2013), size is related to a number of life history properties and also affects the susceptibility of the species to population decline and extinction (Dirzo et al., 2014; Schleuning et al., 2014). This size‐related explanation is reinforced by the importance of body mass in determining the species position of the animal in the models. The mammal species in our dataset are much larger than the birds. Therefore mammals are able to swallow and disperse a larger range of seed and fruit sizes without encountering many morphological constraints (Howe, 1986; Guimarães et al. 2008; Jordano, 1995). Yet, according to Optimal Foraging Theory, large mammals are expected to preferentially forage and disperse seeds from large‐fruited plants because they render more energy, making them more profitable (Herrera, 1989). Stevenson, Pineda, and Samper (2005) have identified size‐coupling (i.e., positive relationships between animal and fruit size) in primates, which is a pattern related to a behavioral interaction mechanism. A possible interpretation of our results is that interaction patterns for mammals are behaviorally driven, resulting from preferential foraging for fruits with specific characteristics within the range of potential feeding items.

Conversely, bird gapes are normally much smaller than mammals' mouths, thus reducing the range of fruit sizes birds can eat, and creating a size threshold in their interaction patterns (Githiru, Lens, Bennur, & Ogol, 2002). Our results agree with those of Burns (2013) on a bird–frugivore assemblage in New Zealand showing that, in general, birds not only present a size‐constrained relationship, but also seem to randomly consume the fruits that are smaller than their gape size. In addition, Wheelwright (1985) clearly catalogued the morphological constraints when he found many large fruits scarred by bill marks below fruiting trees (where survival prospects are low). It is also important to note that, even if the gape of a bird is too small to swallow some fruits, it may be able to consume and effectively disperse large fruits that contain small seeds (e.g., Schupp, Jordano, & Gómez, 2010).

Clearly, passive and behavioral mechanisms are not mutually exclusive. Behavioral processes were found to structure some pairwise interactions between fruits and frugivores in British Columbia (Burns, 2006), and other studies have found behavioral size‐coupling in frugivory assemblages in tropical (Wheelwright, 1985) and Mediterranean systems (Herrera, 2002). At the population level, several authors have identified a preferential consumption of either large (Sobral, Larrinaga, & Guitián, 2010b) or small (Rey, Gutieérrez, Alcántara, & Valera, 1997) fruits by birds, depending on their requirements. On the other hand, the interaction patterns of some small mammals may also present size‐mediated morphological constraints. For example, the large fruits of the *Dipteryx alata* (diameter = 45 mm) tree do not fit the mouth of the small six‐banded armadillo *Euphractus sexcinctus* (mouth size = 15.3 mm). Also, several studies have already shown that fruit selection follows a hierarchical decision‐making process (Andrade, Thies, Rogeri, Kalko, & Mello, 2013; Sallabanks, 1993). This indicates that the variables affecting fruit selection change depending on if the individual is deciding which tree to forage on, which fruit to pick or which seed to swallow. Thus, the mechanisms behind each foraging decision may depend on the hierarchical level of interest.

Surprisingly, none of the variables unrelated to size were found to be important drivers of the interaction patterns in our analyses. For example, dietary specialization has been found to be one of the main factors structuring a Kenyan plant–frugivore network (Schleuning et al., 2011). Additionally, highly frugivorous birds normally need to consume a large variety of fruits to be able to reach their daily requirements of proteins and other nutrients (Moermond & Denslow, 1985). However, frugivory degree does not seem to shape the interaction patterns in this network. Moreover, fruit nutritional content was also not found to be one of the main traits determining interaction rules. This pattern is expected under a passive interaction model driven by morphological constraints, such as those found here for birds. Similarly, fruit energetic content was also not found to be influencing interaction rules, not even for mammals, which seem to prefer large fruits rather than highly energetic ones, as would be expected by Optimal Foraging Theory (MacArthur & Pianka, 1966). However, even if the fruits consumed by mammals were not the most energetic, the net energy income per fruit may be higher in large fruits just because of their size. Besides, these variables may not be important at the decision‐making hierarchical level studied here, but their role can increase at other levels (Andrade et al., 2013; Sallabanks, 1993).

Many studies have also identified species abundance as an important driver of the probability of interaction between mutualistic species (Vázquez et al., 2007, 2009). Abundant species tend to interact with more mutualistic partners than rare species and species interactions are determined by a combination of neutral processes driven by abundance, and deterministic processes related to species traits (Vázquez et al., 2007, 2009). Unfortunately, we did not have information on the abundance of all animal and plant species from the network, and part of the unexplained variation found in this study may be determined by local species abundance. Clearly this is an aspect that warrants further research.

Despite the good general performance of our models, there are caveats related to methodological approach used here. In the niche model for instance, we simulate a unimodal dietary pattern determined by the position of resources on a trait axis. Thus, if we consider body size as the main driver of the interactions, our model does not allow for a species to prefer both small and large fruits (and avoid medium‐sized ones), limiting the suite of behavioral mechanisms simulated. One possible alternative would be to increase the dimensions of the niche model (Eklöf et al., 2013; Pires & Guimarães, 2013), allowing a greater range of differences in preferences of the species. Moreover, our models include a simple set of rules that may be enough to reproduce some general network patterns, but the interactions of some species may be determined by the interplay among many traits and the model can be adequate for some, but not necessarily for all species and interactions. The prevalent rule in a community is determined by the proportion of species whose interactions are better described by one or another set of rules. Consequently, in a seed‐dispersal community formed by both mammals and birds, the prevalent rules will be more related to passive mechanisms if the number of birds is larger than the number of mammals (as it happens in our study community) and behavioral if the number of mammals is larger. Finally, our hypotheses focused more on the animal's perspective, while the plants have also been found to be very important in determining interaction patterns (Bronstein 2009). Thus, further research on the plants perspective is needed.

From a conservation science perspective, the detected interaction rules can be used to reconstruct interaction networks in areas where the information on species interactions is not available and the gathering of interaction data is difficult (Pires et al., 2015). To recreate interaction networks representing these communities, one can estimate the probability of interactions using the distribution of parameters of the models found through optimization and the species characteristics. Even if our approach cannot be used to accurately estimate all pairwise interactions, it can effectively reproduce the overall structure of the interaction networks. In fact, because interactions are labile in nature, changing over time and space, some level of inaccuracy at species level is a desirable property of probabilistic models, as they allow reproducing networks that retain a realistic structure while encompassing our uncertainty on the interactions that do occur.

## Conflict of Interest

The authors declare that they have no conflict of interest.

## Author contribution statement

ESG, PRG, and RD conceived the idea. CD and RD designed the field data gathering. CD performed the fieldwork. MMP wrote Matlab codes; ESG analyzed the data. ESG wrote the manuscript; all authors edited and commented on the manuscript and provided editorial advice.

## References

[ece32865-bib-0001] Andrade, T. Y. , Thies, W. , Rogeri, P. K. , Kalko, E. K. V. , & Mello, M. A. R. (2013). Hierarchical fruit selection by Neotropical leaf‐nosed bats (Chiroptera: Phyllostomidae). Journal of Mammalogy, 94, 1094–1101. doi:10.1644/12‐MAMM‐A‐244.1

[ece32865-bib-0002] Bascompte, J. , Jordano, P. , & Olesen, J. M. (2006). Asymmetric coevolutionary networks facilitate biodiversity maintenance. Science, 312, 431–433. doi:10.1126/science.1123412 16627742 10.1126/science.1123412

[ece32865-bib-0003] Bezerra, E. L. S. , Machado, I. C. , & Mello, M. A. R. (2009). Pollination networks of oil‐flowers: A tiny world within the smallest of all worlds. Journal of Animal Ecology, 78, 1096–1101. doi:10.1111/j.1365‐2656.2009.01567.x 19515098 10.1111/j.1365-2656.2009.01567.x

[ece32865-bib-0501] Bronstein, J. L. (2009). The evolution of facilitation and mutualism. Journal of Ecology, 97, 1160–1170.

[ece32865-bib-0004] Burgos, E. , Ceva, H. , Perazzo, R. P. J. , Devoto, M. , Medan, D. , Zimmermann, M. , & Delbue, A. M. (2007). Why nestedness in mutualistic networks? Journal of Theoretical Biology, 249, 307–313. doi:10.1007/s11538‐013‐9896‐4 17897679 10.1016/j.jtbi.2007.07.030

[ece32865-bib-0005] Burns, K. C. (2006). A simple null model predicts bird‐fruit interactions in a temperate rainforest. Oikos, 115, 427–432. doi:10.1111/j.2006.0030‐1299.15068.x

[ece32865-bib-0006] Burns, K. C. (2013). What causes size coupling in fruit‐frugivore interaction webs? Ecology, 94, 295–300. doi:10.1890/12‐1161.1 23691648 10.1890/12-1161.1

[ece32865-bib-0007] Cazetta, E. , Schaeffer, U. M. , & Galetti, M. (2008). Does attraction to frugivores or defense against pathogens shape fruit pulp composition? Oecologia, 155, 277–286. doi:10.1007/s00442‐007‐0917‐6 18043946 10.1007/s00442-007-0917-6

[ece32865-bib-0008] Chamberlin, T. C. (1890). The method of multiple working hypotheses. Science, 15, 92–96. (reprinted in Science 148:754–759 [1965]). 10.1126/science.148.3671.754 17748786

[ece32865-bib-0009] Cohen, J. E. (1990). A stochastic‐theory of community food webs 0.6. Heterogeneous alternatives to the Cascade model. Theoretical Population Biology, 37, 55–90.

[ece32865-bib-0010] Dehling, D. M. , Jordano, P. , Schaefer, H. M. , Böhning‐Gaese, K. , & Schleuning, M. (2016). Morphology predicts species' functional roles and their degree of specialization in plant–frugivore interactions. Proceedings of the Royal Society B: Biological Sciences, 283, 20152444. 26817779 10.1098/rspb.2015.2444 PMC4795026

[ece32865-bib-0011] Del Hoyo, J. , Elliot, A. , & Sargatal, J. (1992). Handbook of the birds of the world. Barcelona: Lynx Ediciones.

[ece32865-bib-0012] Dirzo, R. , Young, H. S. , Galetti, M. , Ceballos, G. , Isaac, N. J. B. , & Collen, B. (2014). Defaunation in the anthropocene. Science, 345, 401–406. doi:10.1126/science.1251817 25061202 10.1126/science.1251817

[ece32865-bib-0013] Donatti, C. I. (2011) Ecological studies on seed dispersal networks: Insights from a diverse tropical ecosystem, PhD thesis. Stanford University, CA, USA.

[ece32865-bib-0014] Donatti, C. I. , Guimarães, P. R. , Galetti, M. , Pizo, M. A. , Marquitti, F. M. D. , & Dirzo, R. (2011). Analysis of a hyper‐diverse seed dispersal network: modularity and underlying mechanisms. Ecology Letters, 14, 773–781. doi:10.1111/j.1461‐0248.2011.01639.x 21699640 10.1111/j.1461-0248.2011.01639.x

[ece32865-bib-0015] Eklöf, A. , Jacob, U. , Kopp, J. , Bosch, J. , Castro‐Urgal, R. , Chacoff, N. P. , … Allesina, S. (2013). The dimensionality of ecological networks. Ecology Letters, 16, 577–583. doi:10.1111/ele.12081 23438174 10.1111/ele.12081

[ece32865-bib-0016] Elliott, L. P. , & Brook, B. W. (2007). Revisiting Chamberlin: multiple working hypotheses for the 21st century. BioScience, 57, 608–614. doi:10.1641/B570708

[ece32865-bib-0017] Eriksson, O. , & Ehrlén, J. (1991). Phenological variation in fruit characteristics in vertebrate‐dispersed plants. Oecologia, 86, 463–470. doi:10.1007/BF00318311 28313326 10.1007/BF00318311

[ece32865-bib-0018] Galetti, M. , Guevara, R. , Cortes, M. C. , Fadini, R. , Von Matter, S. , Leite, A. B. , … Jordano, P. (2013). Functional extinction of birds drives rapid evolutionary changes in seed size. Science, 340, 1086–1090. doi:10.1126/science.1233774 23723235 10.1126/science.1233774

[ece32865-bib-0502] Guimarães, P. R. , Galetti, M. , & Jordano, P. (2008). Seed dispersal anachronisms: rethinking the fruits extinct megafauna ate. PLoS One, 3, e1745. 18320062 10.1371/journal.pone.0001745 PMC2258420

[ece32865-bib-0019] Githiru, M. , Lens, L. , Bennur, L. A. , & Ogol, C. P. K. O. (2002). Effects of site and fruit size on the composition of avian frugivore assemblages in a fragmented Afrotropical forest. Oikos, 96, 320–330. doi:10.1034/j.1600‐0706.2002.960214.x

[ece32865-bib-0020] Herrera, C. M. (1989). Frugivory and seed dispersal by carnivorous mammals, and associated fruit characteristics, in undisturbed Mediterranean habitats. Oikos, 55, 250–262. doi:10.2307/3565429

[ece32865-bib-0021] Herrera, C. M. (2002). Seed dispersal by vertebrates In Herrera C. M., & Pellmyr O. (Eds.), Plant–animal interactions: an evolutionary approach (pp. 185–208). Oxford: Blackwell.

[ece32865-bib-0022] Howe, H. F. (1986). Seed dispersal by fruit‐eating birds and mammals In Murray D. R. (Ed.), Seed dispersal (pp. 123–189). Sydney: Academic Press Australia.

[ece32865-bib-0023] Jordano, P. (1995). Angiosperm fleshy fruits and seed dispersers: a comparative analysis of adaptation and constraints in plant‐animal interactions. The American Naturalist, 145, 163–191. doi:10.1086/285735

[ece32865-bib-0024] Jordano, P. (2000). Fruits and frugivory In Fenner M. (Ed.), Seeds: the ecology of regeneration in plant communities, 2nd ed. Wallingford: CABI Publisher.

[ece32865-bib-0025] Jordano, P. , Bascompte, J. , & Olesen, J. M. (2003). Invariant properties in coevolutionary networks of plant‐animal interactions. Ecology Letters, 6, 69–81. doi:10.1046/j.1461‐0248.2003.00403.x

[ece32865-bib-0026] Jordano, P. , & Schupp, E. W. (2000). Seed disperser effectiveness: the quantity component and patterns of seed rain for *Prunus mahaleb* . Ecological Monographs, 70, 591–615. doi:10.1890/0012-9615(2000)070[0591:SDETQC]2.0.CO;2

[ece32865-bib-0027] Kirkpatrick, S. , Gelatt, C. D. , & Vecchi, M. P. (1983). Optimization by simulated annealing. Science, 220, 671–680. 17813860 10.1126/science.220.4598.671

[ece32865-bib-0028] MacArthur, R. H. , & Pianka, E. R. (1966). On optimal use of a patchy environment. The American Naturalist, 100, 603–609.

[ece32865-bib-0029] Martinez, I. , Garcia, D. , & Obeso, J. R. (2007). Allometric allocation in fruit and seed packaging conditions: The conflict among selective pressures on seed size. Evolutionary Ecology, 21, 517–533. doi:10.1007/s10682‐006‐9132‐x

[ece32865-bib-0030] MATLAB (2012). The MathWorks Inc., Natick, Massachusetts, USA.

[ece32865-bib-0031] Mckay, M. D. , Beckman, R. J. , & Conover, W. J. (1979). A comparison of three methods for selecting values of input variables in the analysis of output from a computer code. Technometrics, 21, 239–245.

[ece32865-bib-0032] Mello, M. A. R. , Costa, L. F. , Rodrigues, F. , Marquitti, F. M. D. , Sekercioglu, C. , Kissling, D. , & Kalko, E. K. V. (2015). Keystone species in seed dispersal networks are mainly determined by dietary specialization. Oikos, 124, 1031–1039. doi:10.1111/oik.01613

[ece32865-bib-0033] Mello, M. A. R. , Marquitti, F. , Guimarães, P. Jr. , Kalko, E. , Jordano, P. , & de Aguiar, M. (2011). The modularity of seed dispersal: Differences in structure and robustness between bat– and bird–fruit networks. Oecologia, 167, 131–140. 21479592 10.1007/s00442-011-1984-2

[ece32865-bib-0034] Moermond, T. C. , & Denslow, J. S. (1985). Neo‐tropical avian frugivores: patterns of behavior, morphology, and nutrition with consequences for fruit selection. Ornithol Monogr, 36, 865–897.

[ece32865-bib-0035] Olesen, J. M. , Bascompte, J. , Dupont, Y. L. , Erberling, H. , Rasmussen, C. , & Jordano, P. (2011). Missing and forbidden links in mutualistic networks. Proceedings of the Royal Society, 278, 725–732. doi:10.1098/rspb.2010.1371 10.1098/rspb.2010.1371 PMC3030842 20843845

[ece32865-bib-0036] Pires, M. M. , & Guimarães, P. R. (2013). Interaction intimacy organizes networks of antagonistic interactions in different ways. Journal of The Royal Society Interface, 10, 20120649. doi:10.1098/rsif.2012.0649 10.1098/rsif.2012.0649 PMC3565795 23015523

[ece32865-bib-0037] Pires, M. M. , Koch, P. L. , Fariña, R. A. , Aguiar, M. A. M. , dos Reis, S. F. , & Guimarães, P. R. (2015). Pleistocene megafaunal interaction networks became more vulnerable after human arrival. Proceedings of the Royal Society B, 282, 20151367. doi:10.1098/rspb.2015.1367 26336175 10.1098/rspb.2015.1367 PMC4571700

[ece32865-bib-0038] Platt, J. R. (1964). Strong inference. Science, 146, 347–353. 17739513 10.1126/science.146.3642.347

[ece32865-bib-0039] R Development Core Team (2014). R: A language and environment for statistical computing. Vienna, Austria: R Foundation for Statistical Computing.

[ece32865-bib-0040] Rey, P. J. , Gutieérrez, J. E. , Alcántara, J. M. , & Valera, F. (1997). Fruit size in wild olives: Implications for avian seed dispersal. Functional Ecology, 11, 611–618. doi:10.1046/j.1365‐2435.1997.00132.x

[ece32865-bib-0041] Sallabanks, R. (1993). Hierarchical mechanisms of fruit selection by an avian frugivore. Ecology, 74, 1326–1336. doi:10.2307/1940063

[ece32865-bib-0042] Santamaría, L. , & Rodríguez‐Gironés, M. A. (2007). Linkage Rules for Plant‐Pollinator Networks: Trait Complementarity or Exploitation Barriers? PLoS Biology, 5(2), e31. doi:10.1371/journal.pbio.0050031 17253905 10.1371/journal.pbio.0050031 PMC1779813

[ece32865-bib-0043] Sarmento, R. , Alves‐Costa, C. P. , Ayub, A. , & Mello, M. A. R. (2014). Partitioning of seed dispersal services between birds and bats in a fragment of the Brazilian Atlantic Forest. Zoologia, 31, 245–255.

[ece32865-bib-0044] Schleuning, M. , Blüthgen, N. , Flörchinger, M. , Braun, J. , Schaefer, H. M. , & Böhning‐Gaese, K. (2011). Specialization and interaction strength in a tropical plant–frugivore network differ among forest strata. Ecology, 92, 26–36. 21560673 10.1890/09-1842.1

[ece32865-bib-0045] Schleuning, M. , Fründ, J. , & García, D. (2015). Predicting ecosystem functions from biodiversity and mutualistic networks: An extension of trait‐based concepts to plant–animal interactions. Ecography, 38, 380–392. doi:10.1111/ecog.00983

[ece32865-bib-0046] Schleuning, M. , Ingmann, L. , Strauß, R. , Fritz, S. , Dalsgaard, B. , Dehling, D. M. , … Cormann, C. F. (2014). Ecological, historical and evolutionary determinants of modularity in weighted seed‐dispersal networks. Ecology Letters, 17, 454–463. doi:10.1111/ele.12245 24467289 10.1111/ele.12245

[ece32865-bib-0047] Schupp, E. W. , Jordano, P. , & Gómez, J. M. (2010). Seed dispersal effectiveness revisited: A conceptual review. New Phytologist, 188, 333–353. 20673283 10.1111/j.1469-8137.2010.03402.x

[ece32865-bib-0048] Sobral, M. , Larrinaga, A. R. , & Guitián, J. (2010a). Fruit‐size preferences in wild and naive Eurasian blackbirds (*Turdus merula*) feeding on one seed hawthorn (*Crataegus monogyna*). The Auk, 127, 532–539. doi:10.1525/auk.2010.09079

[ece32865-bib-0049] Sobral, M. , Larrinaga, A. R. , & Guitián, J. (2010b). Do seed‐dispersing birds exert selection on optimal plant trait combinations? Correlated phenotypic selection on the fruit and seed size of hawthorn (*Crataegus monogyna*). Ecology and Evolution, 24, 1277–1290. doi:10.1007/s10682‐010‐9380‐7

[ece32865-bib-0050] Stevenson, P. R. , Pineda, M. , & Samper, T. (2005). Influence of seed size on dispersal patterns of woolly monkeys (*Lagothrix lagothricha*) at Tinigua Park, Colombia. Oikos, 110, 435–440. doi:10.1111/j.0030‐1299.2005.12898.x

[ece32865-bib-0051] Tewksbury, J. J. , & Nabhan, G. P. (2001). Directed deterrence by capsaicin in chillies. Nature, 412, 403–404. doi:10.1038/35086653 11473305 10.1038/35086653

[ece32865-bib-0052] Vandekerckhove, J. (2008) General simulated annealing algorithm. http://www.mathworks.de/matlabcentral/fileexchange/10548

[ece32865-bib-0053] Vázquez, D. P. , Blüthgen, N. , Cagnolo, L. , & Chacoff, N. P. (2009). Uniting pattern and process in plant‐animal mutualistic networks: A review. Annals of Botany, 103, 1445–1457. doi:10.1093/aob/mcp057 19304996 10.1093/aob/mcp057 PMC2701748

[ece32865-bib-0054] Vázquez, D. P. , Melián, C. J. , Williams, N. M. , Blüthgen, N. , Krasnov, B. R. , & Poulin, R. (2007). Species abundance and asymmetric interaction strength in ecological networks. Oikos, 116, 1120–1127. doi:10.1111/j.0030‐1299.2007.15828.x

[ece32865-bib-0055] Vidal, M. , Pires, M. M. , & Guimarães, P. R. (2013). Large vertebrates as the missing components of seed dispersal networks. Biological Conservation, 163, 42–48. doi:10.1016/j.biocon.2013.03.025

[ece32865-bib-0056] Wheelwright, N. T. (1985). Fruit size, gape width, and the diets of fruit‐eating birds. Ecology, 66, 808–818.

[ece32865-bib-0057] Williams, R. J. , Anandanadesan, A. , & Purves, D. (2010). The probabilistic Niche model reveals the niche structure and role of body size in a complex food web. PLoS ONE, 5, e12092. doi:10.1371/journal.pone.0012092 20711506 10.1371/journal.pone.0012092 PMC2918514

[ece32865-bib-0058] Williams, R. J. , & Purves, D. W. (2011). The probabilistic Niche model reveals substantial variation in the Niche structure of empirical food webs. Ecology, 92, 1849–1857. doi:10.1890/11‐0200.1 21939081 10.1890/11-0200.1

[ece32865-bib-0059] Woodward, G. , Ebenman, B. , Emmerson, M. , Montoya, J. M. , Olesen, J. M. , Valido, A. , & Warren, P. H. (2005). Body size in ecological networks. Trends in Ecology & Evolution, 20, 402–409. 16701403 10.1016/j.tree.2005.04.005

[ece32865-bib-0060] Zamora, R. (2000). Functional equivalence in plant–animal interactions: Ecological and evolutionary consequences. Oikos, 88, 442–447. doi:10.1034/j.1600‐0706.2000.880222.x

