# Supplementary material for: Species traits and interaction rules shape a species‐rich seed‐dispersal interaction network
Source: Ecol Evol. 2017 May 17;7(12):4496–506. doi: 10.1002/ece3.2865 (PMC5478084; doi:10.1002/ece3.2865)
Supplement: Supplementary file 1 [file ECE3-7-4496-s001.doc]

**Electronic Supplementary Material**

Sebastián-González et al. Species traits and interaction rules shape a species-rich seed dispersal interaction network

This file contains the following supplementary tables and figures:

**Figure S1.** Relationship among the proportion of correctly predicted links and the seed diameter for a) the entire community, b) mammals only and c) birds only. We also show the regression that better fit the data (linear for birds and the entire matrix and polynomial for mammals).

**Figure S2.** Relationship among the proportion of correctly predicted links and the number of links a) & b) the entire community, c) & d) mammals only and e) & f) birds only. We also show the linear regression of the data.

**Table S1.** List of the species included in this study

**Table S2.** List of the species measured in the Museum of Vertebrate Zoology

**Table S3.** AIC values of the simulation where the parameters of the model where constrained to reflect the order of the species depending on their characteristics. Results including both mammals and birds.

**Table S4.** AIC values of the simulation where the parameters of the model where constrained to reflect the order of the species depending on their characteristics. Results including only birds.

**Table S5.** AIC values of the simulation where the parameters of the model where constrained to reflect the order of the species depending on their characteristics. Results including only mammals.

**Appendix S1.** Matlab codes for the modeling approach

**Appendix S2.** R code for the GLM analyses

**Figure S1.** Relationship among the proportion of correctly predicted links and the seed diameter for a) the entire community, b) mammals only and c) birds only. We also show the regression that better fit the data (linear for birds and the entire matrix and polynomial for mammals).

**Figure S2.** Relationship among the proportion of correctly predicted links and the number of links for a) & b) the entire community, c) & d) mammals only and e) & f) birds only. We also show the linear regression of the data.

**Table S1.** List of the species used in this study

| **Species** | **Group** |
| --- | --- |
| *Geochelone carbonaria* | Reptile |
| *Agouti paca* | Mammal |
| *Alouatta caraya* | Mammal |
| *Cerdocyon thous* | Mammal |
| *Dasyprocta* | Mammal |
| *Euphractus sexcintus* | Mammal |
| *Mazama sp.* | Mammal |
| *Nasua nasua* | Mammal |
| *Pecari tajacu* | Mammal |
| *Procyon cancrivorus* | Mammal |
| *Tapirus terrestris* | Mammal |
| *Tayassu pecari* | Mammal |
| *Piaractus mesopotamicus* | Fish |
| *Aratinga aurea* | Bird |
| *Aratinga leucophthalmus* | Bird |
| *Brotogeris versicolurus* | Bird |
| *Casiornis rufa* | Bird |
| *Colomba sp.* | Bird |
| *Crax fasciolata* | Bird |
| *Crypturellus sp* | Bird |
| *Cyanocorax chrysops* | Bird |
| *Cyanocorax cyanomelas* | Bird |
| *Euphonia chlorotica* | Bird |
| *Gnorimopsar chopi* | Bird |
| *Guira guira* | Bird |
| *Icterus croconotus* | Bird |
| *Myiarchus ferox* | Bird |
| *Myiodynastes maculatus* | Bird |
| *Ortalis canicollis* | Bird |
| *Paroaria coronata* | Bird |
| *Pipile pipile* | Bird |
| *Pitangus sulphuratus* | Bird |
| *Psarocolis decumanus* | Bird |
| *Pteroglossus castanotis* | Bird |
| *Ramphastos toco* | Bird |
| *Ramphocelus carbo* | Bird |
| *Rhea americana* | Bird |
| *Saltator coerulescens* | Bird |
| *Tachyphonus rufus* | Bird |
| *Thraupis palmerum* | Bird |
| *Thraupis sayaca* | Bird |
| *Tityra cayana* | Bird |
| *Trogon curucui* | Bird |
| *Turdus rufiventris* | Bird |
| *Tyrannus melancholicus* | Bird |
| *Acrocomia aculeata* | Plant |
| *Agonandra brasiliensis* | Plant |
| *Alibertia sessilis* | Plant |
| *Annona dioica* | Plant |
| *Attalea phalerata* | Plant |
| *Bactris glaucescens* | Plant |
| *Byrsonima orbigniana* | Plant |
| *Byrsonima verbascifolia* | Plant |
| *Caryocar brasiliensis* | Plant |
| *Cecropia pachystachia* | Plant |
| *Copernicia alba* | Plant |
| *Couepia uiti* | Plant |
| *Curatella americana* | Plant |
| *Diospyrus hispida* | Plant |
| *Dipteryx alata* | Plant |
| *Doliocarpus dentatus* | Plant |
| *Dulacea egeri* | Plant |
| *Enterolobium contortisiliquum* | Plant |
| *Eugenia desynterica* | Plant |
| *Ficus gomelleira* | Plant |
| *Ficus pertusa* | Plant |
| *Garcinia brasiliensis* | Plant |
| *Genipa americana* | Plant |
| *Guazuma ulmifolia* | Plant |
| *Hancornia speciosa* | Plant |
| *Hymenaea stigonocarpa* | Plant |
| *Inga laurina* | Plant |
| *Licania parvifolia* | Plant |
| *Meliococcus lepidopetalius* | Plant |
| *Mouriri elliptica* | Plant |
| *Ocotea dyospirifolia* | Plant |
| *Phoradendron* | Plant |
| *Pouteria gardneri* | Plant |
| *Pouteria ramiflora* | Plant |
| *Protium heptaphyllum* | Plant |
| *Psidium nutans* | Plant |
| *Psitacanthus calliculatus* | Plant |
| *Psitacanthus cordatus* | Plant |
| *Rhamnidium elaeocarpum* | Plant |
| *Salacia elliptica* | Plant |
| *Sterculia apetala* | Plant |
| *Swartia jorori* | Plant |
| *Syagrus sp.* | Plant |
| *Tocoyena sp.* | Plant |
| *Vitex cymosa* | Plant |
| *Zanthoxyllum rigidum* | Plant |

**Table S2.** List of the species measured in the Museum of Vertebrate Zoology, University of California Berkeley. We show the mean values of the mouth or of beak size measured and the number of specimens measured.

| **﻿Species** | **Group** | **Mouth (mm)** | **Beak (mm)** | **N** |
| --- | --- | --- | --- | --- |
| *Casiornis rufa* | bird |  | 7.5 | 1 |
| *Cyanocorax chrysops* | bird |  | 14.2 | 4 |
| *Cyanocorax cyanomelas* | bird |  | 16.8 | 4 |
| *Gnorimopsar chopi* | bird |  | 10.5 | 2 |
| *Guira guira* | bird |  | 10.7 | 3 |
| *Myiarchus ferox* | bird |  | 10.4 | 5 |
| *Paroaria coronata* | bird |  | 9.7 | 5 |
| *Thraupis palmarum* | bird |  | 9.1 | 7 |
| *Agouti paca* | mammal | 8.3 |  | 5 |
| *Cerdocyon thous* | mammal | 12.2 |  | 5 |
| *Euphractus sexcinctus* | mammal | 9.4 |  | 2 |
| *Mazama americana* | mammal | 18.6 |  | 5 |
| *Nasua nasua* | mammal | 11 |  | 2 |
| *Pecari tajacu* | mammal | 8.3 |  | 4 |
| *Procyon cancrivorus* | mammal | 14.3 |  | 5 |
| *Tapirus terrestris* | mammal | 33.1 |  | 4 |
| *Tayassu pecari* | mammal | 8 |  | 1 |

**Table S3.** AIC values of the simulation where the parameters of the model were constrained to reflect the order of the species depending on their characteristics. Results for the matrix including both mammals and birds. The value in bold identifies the model with the best fit (lowest AIC).

|  | Cascade | | Niche | | | TNM | | | |
| --- | --- | --- | --- | --- | --- | --- | --- | --- | --- |
|  | *n_P_* | *n_A_* | *n_P_* | *c_A_* | *r_A_* | *n_A_* | *n_P_* | *c_A_* | *r_A_* |
| Frugivory |  | 1970.6 |  | 1988.8 | 2071.4 | 2358.5 |  | 2087.8 | 1952.4 |
| Body mass |  | 2039.5 |  | 1990.4 | 1963.7 | 2086.4 |  | 2068.2 | 2464 |
| Fruit Diameter | **1775.6** |  | 2027.9 |  |  |  | 2185.5 |  |  |
| Seed diameter | 1812 |  | 2097.74 |  |  |  | 2268.6 |  |  |

**Table S4.** AIC values of the simulation where the parameters of the model were constrained to reflect the order of the species depending on their characteristics. Results for the matrix including only birds. The value in bold identifies the model with the best fit (lowest AIC).

|  | Cascade | | Niche | | | TNM | | | |
| --- | --- | --- | --- | --- | --- | --- | --- | --- | --- |
|  | *n_P_* | *n_A_* | *n_P_* | *c_A_* | *r_A_* | *n_A_* | *n_P_* | *c_A_* | *r_A_* |
| Frugivory |  | 1023.8 |  | 1220 | 1290.1 | 1735.6 |  | 1539.9 | 1833.6 |
| Body mass |  | 1018 |  | 1201.4 | 1305.6 | 1655.2 |  | 1860.3 | 1254.3 |
| Fruit Diameter | **1014.7** |  | 1154 |  |  |  | 1340.4 |  |  |
| Seed diameter | 1025.0 |  | 1178.8 |  |  |  | 1342.8 |  |  |

**Table S5.** AIC values of the simulation where the parameters of the model were constrained to reflect the order of the species depending on their characteristics. Results for the matrix including only mammals. The value in bold identifies the model with the best fit (lowest AIC).

|  | Cascade | | Niche | | | TNM | | | |
| --- | --- | --- | --- | --- | --- | --- | --- | --- | --- |
|  | *n_P_* | *n_A_* | *n_P_* | *c_A_* | *r_A_* | *n_A_* | *n_P_* | *c_A_* | *r_A_* |
| Frugivory |  | 822.1 |  | 948.4 | 799.7 | 980.9 |  | 1011.8 | 980.7 |
| Body mass |  | **681.4** |  | 793.8 | 818.7 | 911.5 |  | 868.0 | 1141.6 |
| Fruit Diameter | 967.8 |  | 1276 |  |  |  | 1463.6 |  |  |
| Seed diameter | 946.1 |  | 1241.5 |  |  |  | 1994.5 |  |  |

**Appendix S1.** Matlab code for the modeling approach

**File 1: MLEmodels**

% Species traits and interaction rules shape a species-rich seed dispersal interaction network Sebastián-González et al.

% 26-06-2013 - Script created

% Finding MLE for niche-cascade and truncated niche models

% ============================================================

clear all

clc

matrix=dlmread('B2.txt');%reading matrix

% matrix=ordena(matrix);

[m,n]=size(matrix);

C=sum(matrix(:))/(m*n);%connectance

S=m+n;

% ------------------------------------------------

% Latin Hypercube

% Samples initial values for the free parameters after subdividing the parameter space

% ------------------------------------------------

intervs=0:0.05:1; %smaller steps can be used for more replicates

rep=length(intervs)-1;

% Cascade

vec_matC=zeros((S),rep);

for i=1:(S)

lathc=randsample(length(intervs)-1,length(intervs)-1); %generates sampling intervals

low=intervs(lathc(1:rep)); %lower limit of each interval

upp=low+0.05; %upper limit

vec_matC(i,:)=low + (upp-low).* rand(1,rep);%sampling a value within the interval

end

% vec_mat is a matrix with initial guesses

% rows = guess values for a given parameters

% columns = each parameter vector

% Niche

vec_matN=zeros((S+m),rep);

for i=1:(S+m)

lathc=randsample(length(intervs)-1,length(intervs)-1); %generates sampling intervals

low=intervs(lathc(1:rep)); %lowere limit of each interval

upp=low+0.05; %upper limit

vec_matN(i,:)=low + (upp-low).* rand(1,rep); %matrix with initial guess - rows = guess values for a given parameters ; columns = each parameter vector

end

% truncated

vec_matT=zeros((S+2*m),rep);

for i=1:(S+2*m)

lathc=randsample(length(intervs)-1,length(intervs)-1); %generates sampling intervals

low=intervs(lathc(1:rep)); %lowere limit of each interval

upp=low+0.05; %upper limit

vec_matT(i,:)=low + (upp-low).* rand(1,rep); %matrix with initial guess - rows = guess values for a given parameters ; columns = each parameter vector

end

% -----------------------------

% Finding MLE

% -----------------------------

% Defining loss function

lossC = @(vec)LLCascade(vec);

lossN = @(vec)LLNiche_2(vec);

lossT = @(vec)LLTruncated_niche(vec);

rep=length(intervs)-1;

mle_matC=zeros((S),rep);

mle_matN=zeros((S+m),rep);

mle_matT=zeros((S+2*m),rep);

ll_mat=zeros(3,rep);

for k=1:rep

vecC=vec_matC(:,k)';

[mleC llC]=anneal_mod(lossC,vecC);%the parameters of anneal function can be tweaked to ease convergence

vec_matC(:,k)=vecC;%initial guess of parameters

mle_matC(:,k)=mleC;%parameters after optimization

ll_mat(1,k)=llC;%AIC values

k

llC

end

for k=1:rep

vecN=vec_matN(:,k)';

[mleN llN]=anneal_mod(lossN,vecN);

vec_matN(:,k)=vecN;%initial guess of parameters

mle_matN(:,k)=mleN;%parameters after optimization

ll_mat(2,k)=llN;%AIC values

k

llN

end

for k=1:rep

vecT=vec_matT(:,k)';

[mleT llT]=anneal_mod(lossT,vecT);

vec_matT(:,k)=vecT;%initial guess of parameters

mle_matT(:,k)=mleT;%parameters after optimization

ll_mat(3,k)=llT;%AIC values

k

llT

end

dlmwrite('aic.B2.txt',ll_mat,'\t')

dlmwrite('mlecascade.B2.txt',mle_matC,'\t')

dlmwrite('mleniche.B2.txt',mle_matN,'\t')

dlmwrite('mletruncated.B2.txt',mle_matT,'\t')

**File 2: LLCascade**

% 30-11-2011 - Script created

% Logit based Cascade model

% ===========================================================

function AIC = LLCascade(nvec)

%-----------------------------------------------%

%1. Importing data and defining variables

%-----------------------------------------------%

mat=dlmread('B2.txt');

% mat=matrix;

[m,n]=size(mat);

S=m+n;

%----------------------------------------------------------%

%2.Assigning niche position (ni) for predator and prey (nj)

%---------------------------------------------------------%

%npred=nvec(1:m); %If c and r are defined a priori there is no need for

%predators positioning. They just need diet centers and ranges as needed

%for P - a fortunate feature of two-mode nets

nprey=nvec(1:n);

npred=nvec((n+1):S);

% ----------------

% logit regression

% ---------------

% table

tab=zeros(S,2);

counter=1;

for i=1:m

for j=1:n

tab(counter,1)=mat(i,j); %tab first column=matrix value

if npred(i)>nprey(j)

tab(counter,2)=1; %tab second column 1 if npred>nprey, 0 otherwise

else

tab(counter,2)=0;

end

counter=counter+1;

end

end

% regression

N=ones(m*n,1);

X=[ones(m*n,1) tab(:,2)]; %constant vector plus the covariate

log_par=logist(tab(:,1),N,X); %mle of logit regression parameters. Needs statbox

%-----------------------------------------------%

% Interaction probability matrix

%-----------------------------------------------%

% Guess for logit parameters

% alpha=nvec(S+1);

% beta=nvec(S+2);

alpha=log_par(1);

beta=abs(log_par(2)); %beta should be a positive value

Pmat=zeros(m,n);

for i=1:m

for j=1:n

if npred(i)>nprey(j)

aux=(exp(alpha+beta*1)); %probability when npred>nprey

else

aux=(exp(alpha+beta*0));

end

Pmat(i,j)= aux/(1+aux) ; %logit function

end

end

Pmat=Pmat+1e-010; %avoid P=0

%-----------------------------------------------%

% Log Likelihood matrix

%-----------------------------------------------%

logP=log(Pmat); %Log of Pmat

logPinv=log(1-Pmat); %log of (1-Pmat)

LLmat= zeros(m,n);

for i=1:m

for j=1:n

LLmat(i,j)=(mat(i,j)*logP(i,j))+((1-mat(i,j))*logPinv(i,j)); %(Aij*p)+(1-Aij*(1-p))

end

end

%-----------------------------------------------%

% Negative Log Likelihood

%-----------------------------------------------%

NLLi=-sum(LLmat(:));

AIC=2*S+2*NLLi; %AIC of the model 2k+2*NLL, where k=parameters

**File 3: LLNiche_2**

% 29-10-2011 - Script created

% Probabilistic niche model (Williams 2010; Williams and Purves 2011)

% version 2 center of diet (ci) and range (ri) are free parameters

% ==========================================================

function AIC = LLNiche(nvec)

%-----------------------------------------------%

%1. Importing data and defining variables

%-----------------------------------------------%

mat=dlmread('B2.txt');

% mat=matrix;

[m,n]=size(mat);

S=m+n;

%----------------------------------------------------------%

%2.Assigning niche position (ni) for predator and prey (nj)

%---------------------------------------------------------%

%npred=nvec(1:m); %If c and r are defined a priori there is no need for

%predators positioning. They just need diet centers and ranges as needed

%for P - a fortunate feature of two-mode nets

nprey=nvec(1:n);

%-------------------------------------------------------------------

%3. Assigning diet center (ci) and ranges (ri) for predators (rows)

%-------------------------------------------------------------------

cvalues=nvec((n+1):S);

rvalues=nvec((S+1):(S+m));

%-----------------------------------------------%

% Interaction probability matrix

%-----------------------------------------------%

Pmat=zeros(m,n);

alpha=1; % Probablity of preying the optimal prey

% e=nvec(m+n+1); %If used as a free parameter

e=2; % modifies function shape (larger values = flatter)

for i=1:m

for j=1:n

aux=(nprey(j)-cvalues(i))/(rvalues(i)/2);

Pmat(i,j)= alpha*(exp(-(aux)^e)) ;%Probability matrix

end

end

Pmat=Pmat+1e-010; %avoid P=0

%-----------------------------------------------%

% Log Likelihood matrix

%-----------------------------------------------%

logP=log(Pmat); %Log of Pmat

logPinv=log(1-Pmat); %log of (1-Pmat)

LLmat= zeros(m,n);

for i=1:m

for j=1:n

LLmat(i,j)=(mat(i,j)*logP(i,j))+((1-mat(i,j))*logPinv(i,j)); %(Aij*p)+(1-Aij*(1-p))

end

end

%-----------------------------------------------%

% Negative Log Likelihood

%-----------------------------------------------%

NLLi=-sum(LLmat(:));

AIC=2*(S+m)+2*NLLi; %AIC of the model 2k+2*NLL, where k=parameters

**File 4: LLTruncated_niche**

% 16-05-2013 - Script created

% Truncated version

% ==========================================================

function AIC = LLTruncated_niche(nvec)

%nvec should be n+3m in length

%-----------------------------------------------%

%1. Importing data and defining variables

%-----------------------------------------------%

mat=dlmread('B2.txt');

% mat=matrix;

[m,n]=size(mat);

S=m+n;

%nvec=rand(1,S+2*m); %Modification for the truncated version

%----------------------------------------------------------%

%2.Assigning niche position (ni) for predator and prey (nj)

%---------------------------------------------------------%

%npred=nvec(1:m); %If c and r are defined a priori there is no need for

%predators positioning. They just need diet centers and ranges as needed

%for P - a fortunate feature of two-mode nets

nprey=nvec(1:n);

npred=nvec((n+1):S); %Predator truncation

%-------------------------------------------------------------------

%3. Assigning diet center (ci) and ranges (ri) for predators (rows)

%-------------------------------------------------------------------

cvalues=nvec((S+1):(m+S));

rvalues=nvec((S+m+1):(S+2*m)); %Modification for the truncated version

%-----------------------------------------------%

% Interaction probability matrix

%-----------------------------------------------%

Pmat=zeros(m,n);

alpha=1; % Probablity of preying the optimal prey

% e=nvec(m+n+1); %If used as a free parameter

e=2; % modifies function shape (larger values = flatter)

for i=1:m

for j=1:n

if nprey(j)<npred(i)

aux=(nprey(j)-cvalues(i))/(rvalues(i)/2);

Pmat(i,j)= alpha*(exp(-(aux)^e)) ;

else

Pmat(i,j)=0.0001;

end

end

end

Pmat=Pmat+1e-010; %avoid P=0

%-----------------------------------------------%

% Log Likelihood matrix

%-----------------------------------------------%

logP=log(Pmat); %Log of Pmat

logPinv=log(1-Pmat); %log of (1-Pmat)

LLmat= zeros(m,n);

for i=1:m

for j=1:n

LLmat(i,j)=(mat(i,j)*logP(i,j))+((1-mat(i,j))*logPinv(i,j)); %(Aij*p)+(1-Aij*(1-p))%%Formula likelihood

end

end

%-----------------------------------------------%

% Negative Log Likelihood

%-----------------------------------------------%

NLLi=-sum(LLmat(:));

AIC=2*(S+2*m)+2*NLLi; %AIC of the model 2k+NLL, where k=parameters

**File 5: logit**

function [beta,mu,dev,df,se]=logist(y,n,x,offset,print);

%LOGIST Fit logistic regression model.

% [BETA,MU,DEV,DF,SE]=LOGIST(Y,N,X,OFFSET,PRINT)

% All input and output arguments except Y are optional.

%

% Y - response vector containing binomial counts

% N - number of trials for each count. Y is assumed to be binomial(p,N).

% X - matrix of covariates, including the constant vector if required

% OFFSET - offset if required

% PRINT - enter any argument if output required each iteration

%

% BETA - regression parameter estimates

% SE - associated standard errors

% MU - fitted values

% DEV - residual deviance

% DF - residual degrees of freedom

% GKS 18 May 2002

% Initialize

y=y(:);

[my ny]=size(y);

if nargin<2, disp('Must specify binomial N'); return; end;

n=n(:);

if (min(n-y)<0), disp('Binomial counts Y must be less than or equal to binomial N'); return; end;

if nargin<3, x=ones(my,ny); end;

[mx nx]=size(x);

if nargin<4, offset=0; end;

% Starting values

y0=y+0.5.*(y==0);

yn=y-0.5.*(y==n);

lp=log((y+0.5)./(n-y+0.5))-offset;

% Iteratively reweighted least squares

dev=1e6; devold=dev+1;

if nargin==5, disp('dev'); end;

while abs(devold-dev) > 1e-8;

p=exp(offset+lp);

p=p./(1+p);

mu=n.*p;

devold=dev;

dev=2.*sum(y.*log(y0./mu) + (n-y).*log((n-yn)./(n-mu)));

v=mu.*(n-mu)./n;

z=(y-mu)./v+lp;

beta=( x'*((v*ones(1,nx)).*x) )\( x'*(v.*z) );

lp=x*beta;

if nargin==5, disp(dev); end;

end;

if nargout>3, df=my-nx; end;

if nargout>4, se=sqrt(diag(inv( x'*((v*ones(1,nx)).*x) ))); end;

**Appendix S2.** R script for GLMs

################################################################################################

#Script for the GLMs

#Esther Sebastian-Gonzalez

#Species traits and interaction rules shape a species-rich seed dispersal interaction network

#Esther Sebastián-González, Mathias M. Pires, Camila I. Donatti, Paulo R. Guimarães Jr, Rodolfo Dirzo

#####################################################################################################

#Read table with position and characteristics animals

read.table(nvec.a1,header=T, sep="\t")->nvec.a1

read.table(nvec.a2,header=T, sep="\t")->nvec.a2 #When the model is niche and there are two parameters

#Read table with position and characteristics plants

read.table(nvec.p, header=T, sep="\t")->nvec.p

#Create a function to perform the randomizations that returns the coefficient and the p-value

aleat=function (x,y)

{

mod1=lm(x~y)

xob=mod1$coefficients[2]

vec<-rep(0,1000)

for(i in 1:1000)

{

xk<-sample(x)

mod<-lm(xk~y)

vec[i]<-mod$coefficients[2]

}

pvalue<-length(vec[vec>=xob])/1000

results<-list(xob, pvalue)

return(results)

}

#Perform analyses for fruits

aleat(nvec.p$order.p , nvec.p$fruitmass)

aleat(nvec.p$order.p , nvec.p$fruitdiameter)

aleat(nvec.p$order.p , nvec.p$seed.mass)

aleat(nvec.p$order.p , nvec.p$seed.diam)

aleat(nvec.p$order.p , nvec.p$energy)

aleat(nvec.p$order.p , nvec.p$fruit_avail)

aleat(nvec.p$order.p , nvec.p$prot2)

aleat(nvec.p$order.p , nvec.p$lipids)

aleat(nvec.p$order.p , nvec.p$carboh_sol)

#Perform analyses for animals

aleat(nvec.a1$order.a1 , nvec.a1$logbodymass)

aleat(nvec.a1$order.a1 , nvec.a1$beak.mouth)

aleat(nvec.a1$order.a1 , nvec.a1$frugivory)

#Perform analyses for animals

aleat(nvec.a2$order.a2 , nvec.a2$logbodymass)

aleat(nvec.a2$order.a2 , nvec.a2$beak.mouth)

aleat(nvec.a2$order.a2 , nvec.a2$frugivory)
